# Identification of Single Nucleotide Polymorphic Loci and Candidate Genes for Seed Germination Percentage in Okra under Salt and No-Salt Stresses by Genome-Wide Association Study

**DOI:** 10.3390/plants13050588

**Published:** 2024-02-22

**Authors:** Gaowen Xu, Yujing Cheng, Xiaoqiu Wang, Zhigang Dai, Zepei Kang, Zhichao Ye, Yangyang Pan, Linkang Zhou, Dongwei Xie, Jian Sun

**Affiliations:** 1 School of Life Sciences, Nantong University, Nantong 226019, China; xugaowen8@163.com (G.X.); 16651073354@163.com (Z.Y.); 18952552326@163.com (Y.P.); zlk321283@163.com (L.Z.); 2 Jiangsu Yanjiang Institute of Agricultural Sciences, Nantong 226019, China; yjcheng_1669@163.com (Y.C.); 15205270412@163.com (X.W.); 3 Institute of Bast Fiber Crops, Chinese Academy of Agricultural Sciences, Changsha 410205, China; dzgmonkey@126.com (Z.D.); kzp18860380256@163.com (Z.K.)

**Keywords:** okra, germination, salt tolerance, GWAS, candidate gene

## Abstract

Excessive soil salinity is a major stressor inhibiting crops’ growth, development, and yield. Seed germination is a critical stage of crop growth and development, as well as one of the most salt-sensitive stages. Salt stress has a significant inhibitory effect on seed germination. Okra is a nutritious vegetable, but its seed germination percentage (GP) is low, whether under salt stress conditions or suitable conditions. In this study, we used 180 okra accessions and conducted a genome-wide association study (GWAS) on the germination percentage using 20,133,859 single nucleotide polymorphic (SNP) markers under 0 (CK, diluted water), 70 (treatment 1, T1), and 140 mmol/L (treatment 2, T2) NaCl conditions. Using the mixed linear model (MLM) in Efficient Mixed-model Association eXpedated (EMMAX) and Genome-wide Efficient Mixed Model Association (GEMMA) software, 511 SNP loci were significantly associated during germination, of which 167 SNP loci were detected simultaneously by both programs. Among the 167 SNPs, SNP2619493 on chromosome 59 and SNP2692266 on chromosome 44 were detected simultaneously under the CK, T1, and T2 conditions, and were key SNP loci regulating the GP of okra seeds. Linkage disequilibrium block analysis revealed that nsSNP2626294 (C/T) in *Ae59G004900* was near SNP2619493, and the amino acid changes caused by nsSNP2626294 led to an increase in the phenotypic values in some okra accessions. There was an nsSNP2688406 (A/G) in *Ae44G005470* near SNP2692266, and the amino acid change caused by nsSNP2688406 led to a decrease in phenotypic values in some okra accessions. These results indicate that *Ae59G004900* and *Ae44G005470* regulate the GP of okra seeds under salt and no-salt stresses. The gene expression analysis further demonstrated these results. The SNP markers and genes that were identified in this study will provide reference for further research on the GP of okra, as well as new genetic markers and candidate genes for cultivating new okra varieties with high GPs under salt and no-salt stress conditions.

## 1. Introduction

Soil salinization is a significant problem facing agriculture, as it seriously affects the sustainable use of some cultivated land [1]. Due to climate change, excessive land reclamation, and industrial pollution, the area of salinized land is continuously increasing [2]. The global area of saline soils has exceeded 800 million hectares, accounting for about 6% of the total global land area. About 20% of the irrigated land worldwide is affected by saline soil [3]. Most crops are sweet-soil species that are not salt-tolerant, and a highly salty environment harms them [4]. Soil salinization is an important environmental factor that affects germination, growth and development, and yield. Soil salinization poses a serious threat to global crop production [5].

Salt stress is usually caused by high concentrations of Na^+^ and Cl^−^ in the soil [6]. The harm to plants includes the pathways of osmotic stress, ionic stress, and secondary stress [4]. When the soil’s salt concentration is too high, the internal osmotic potential of cells increases, while the external environmental osmotic potential is lower. The permeability difference between the internal and external environment leads to osmotic stress, thereby damaging the ability to absorb water and causing water leakage [2]. The excessive accumulation of Na^+^ and Cl^−^ disrupts the ion balance within cells, affects metabolism, and hinders growth and development [7,8]. In addition, reactive oxygen species, such as superoxide radicals (O_2_^−^), hydrogen peroxide (H_2_O_2_), and singlet oxygen (^1^O_2_), accumulate excessively due to salt stress, leading to damage and inhibited normal metabolic activities [9].

The tolerance of plants to salt stress is a quantitative trait that is controlled by multiple genes, which are influenced by environmental factors, making it a complex mechanism [10,11]. The salt tolerance of different plants species or different genotypes of the same plant species often varies. Screening and cultivating new varieties of salt-tolerant plants is an economical and effective way to alleviate the negative effects of salt stress on plants. It is extremely important to study the genetic basis of plants’ salt tolerance mechanisms to develop new salt-tolerant varieties [11]. With the development of high-throughput sequencing technology and the widespread application of SNP markers, genome-wide association studies (GWASs) have become a more effective method for mining plant genes than traditional QTL mapping methods. At present, GWASs based on large-scale SNP markers have been widely used in gene mining for complex traits in plants, such as rice [12], wheat [13], cotton [14], soybean [15], watermelon [16], and cherry [17].

The growth of plants includes seed germination, seedling development, nutrient organ growth, and reproductive organ growth. Among these stages, seed germination is the most significant for completing the entire life process of the plant [18,19]. The salt tolerance of plants varies at different stages of growth and development, and the sensitivity of seed germination to salt stress is higher than that of other developmental stages. Therefore, it is crucial to identify gene loci related to salt tolerance during seed germination [20,21]. Many studies have conducted a GWAS to identify salt-tolerant SNP loci during seed germination in major food and commercially important crops [22,23,24,25]. Ravelombola et al. [26] used 116 cowpea accessions and 1049 SNPs generated by genotyping-by-sequencing to perform a GWAS on seed germination rates. Three SNPs (Scaffold87490_622, Scaffold87490_630, and C35017374_128) were significantly associated with the target traits and were useful as candidate molecular markers for breeding salt-tolerant cowpea.

Okra (*Abelmoschus esculentus* L. Moench) is an annual herb in the mallow family. It was originally classified in the *Hibiscus* genus but was later classified as a separate okra species because of its early calyx fall [27]. The immature fruit of okra is rich in flavonoids, sugars, alkaloids, dietary fiber, iron, calcium, and manganese and has extremely high nutritional value [28]. Studies have shown that okra has several health benefits such as reducing blood sugar and blood lipids [29], protecting the nervous system [30], protecting the liver [31], and fighting cancer [32]. Okra has been featured as a functional and nutritious vegetable and has received increasing attention [33]. Okra is not a salt-tolerant crop, and soil salinity is a partial reason for its lower productivity [34,35]. Unfortunately, there is limited research on the salt tolerance mechanism and salt-tolerant genes in okra, making it very difficult to improve its salt tolerance through molecular breeding. In this study, we used 180 okra accessions to conduct a GWAS on salt tolerance during seed germination, aiming to identify the key salt-tolerant genes during seed germination and provide a reference for cultivating new salt-tolerant varieties of okra using molecular breeding methods.

## 2. Results

### 2.1. Phenotypic Variations

We treated the seeds of the 180 okra accessions with 0 (CK), 70 (T1), or 140 mmol/L (T2) NaCl. On days 3 and 7 after the treatments, the GP was counted, and six sets of phenotypic data were obtained (Table 1). The GP of the 180 accessions under the salt treatment decreased to varying degrees on days 3 and 7 compared with the CK condition, indicating that salt stress inhibits the germination of okra seeds. The germination results on day 3 showed that the GP under the T1 condition was 0.3294, which decreased by 0.3787 compared to the GP under the CK condition, with a decrease of 114.97%. The GP under the T2 condition decreased by 0.5417 compared to the CK condition, with a decrease of 326.12%. The germination results on day 3 showed that the GP under the T1 condition decreased by 0.4134 compared to the CK condition, with a decrease of 105.81%. The GP under the T2 condition decreased by 0.4964 compared to the CK condition, with a decrease of 161.33%. These results show that the overall GP of the 180 selected okra accessions in this study was not high under the CK condition, with a GP-CK-7 d of 0.8041 and a GP-CK-3 d of only 0.7081. The GP of T1 and T2 decreased significantly compared to the CK under the salt condition. The decrease in the T2 treatment was as high as 326.12% on day 3 and 161.33% on day 7, indicating that the GP of okra seeds reached an extremely low level on day 3 after the salt treatment, but the germination ability was restored.

Some okra accessions exhibited higher GPs under both the CK and salt stress conditions, while others exhibited the opposite. The GP of the top ten and bottom ten accessions in each treatment are shown in Table S3. Most of the accessions in Table S3 appeared multiple times in the six traits, indicating that they had stable and high/low GPs under different treatments. Accessions 26, 32, and 99 all ranked in the top ten for all treatments. Accessions 14 and 97 only had a high GP under salt treatments but did not enter the top ten under the CK condition. Accessions 66, 82, and 141 all ranked in the bottom ten in all treatments. Accession 145 only had a low GP under salt treatments but did not enter the bottom ten under the CK condition. The above accessions can be used as important materials for studying the germination of okra seeds under salt and no-salt stresses.

**Figure 1:**
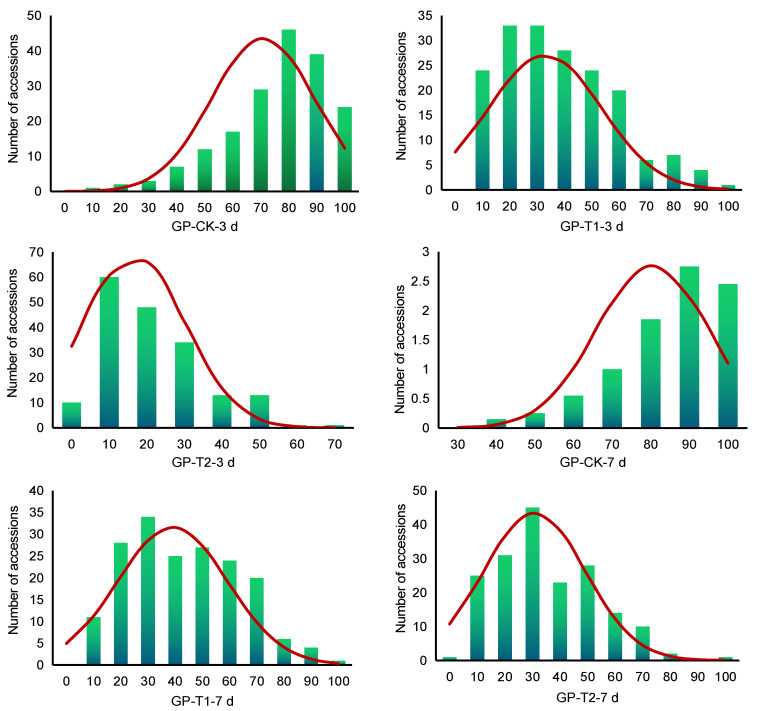
Frequency distribution of GP in 180 okra accessions.

The coefficient of variation of the six traits ranged from 17.96% to 82.07%, indicating larger phenotypic variation. After the salt treatment, the phenotypic variation of the 180 accessions increased (51.76–82.07%), indicating the emergence of more salt-tolerant and salt-sensitive okra accessions during germination, which was very powerful for mining salt-tolerant genes from these accessions. The absolute values of skewness and kurtosis of most traits were less than 1, indicating that the six trait values of the 180 accessions were all normal or near-normal distributions (Figure 1), which conforms to the law of continuous changes in quantitative traits and was suitable for the GWAS.

To understand the relationships between the GPs under CK, T1, and T2 conditions, the correlations between the six traits were analyzed using the mean values of the 180 okra accessions (Table 2). The results showed that except for a significant positive correlation between GP-CK-7 d and GP-T2-3 d, all other traits were highly significantly positively correlated. The correlation coefficients between the GPs under the CK condition (GP-CK-3 d and GP-CK-7 d) and the GPs under salt stress conditions (GP-T1-3 d, GP-T1-7 d, GP-T2-3 d, GP-T2-7 d) were small (0.1825~0.3863), while the correlation coefficients between the GPs under salt stress conditions (GP-T1-3 d, GP-T1-7 d, GP-T2-3 d, GP-T2-7 d) were large (0.5297~0.7069).

### 2.2. Genotyping and Population Structural Analysis

We resequenced the genomes of the 180 okra accessions and generated 8.8 Gb of clean reads. The average coverage depth of a sample was 11.18×, and the Q30 was 94.61%. A total of 20,133,859 high-quality SNPs were obtained. On average, 111,855 SNPs were obtained per accession, with the highest being 467,629 and the lowest being 3117, indicating that these okra accessions have a wide range of SNPs (Table S4).

**Figure 2:**
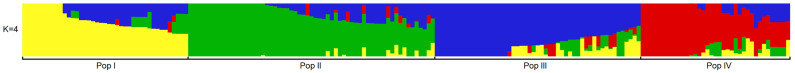
Population structure of 180 okra cultivars and 10 wild species using SNP genotyping data. a: population structure based on K = 4 using the ADMIXTURE program. The *x*-axis represents the okra accessions, and the *y*-axis represents the probability that an individual belongs to a subgroup. Yellow, green, blue, and red represent Pop I, Pop II, Pop III, and Pop IV, respectively.

**Figure 3:**
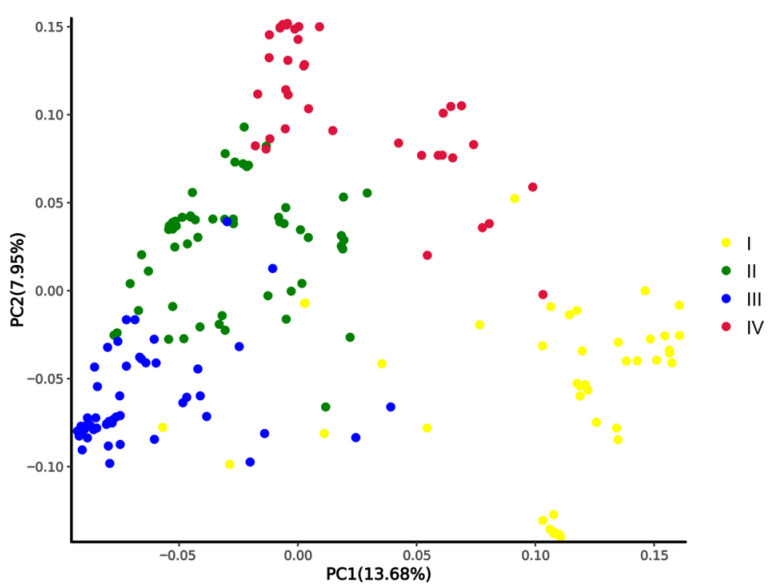
PCA of 180 okra cultivars and 10 wild species. PC1 and PC2 refer to the first and second principal components, respectively. The colors of dots correspond to the structure grouping.

We conducted a population structural analysis on the 180 accessions of okra (*Abelmoschus esculentus*) and 10 wild species of okra (*Abelmoschus manihot*), and the results showed that the 180 cultivated okra and the 10 wild species were divided into four subgroups (Pop I, Pop II, Pop III, and Pop IV) (Figure 2). Pop I had 41 accessions, including 10 wild species and 31 cultivated species, all with five arris. Pop II had 61 accessions, most of which had five arris. Pop III had 51 accessions, most of which had multiple arris (6–9 arris). Pop IV had 37 accessions, most of which had no arris. The results of the PCA and population structure analysis are very similar (Figure 3).

### 2.3. GWAS of GP under CK and Salt Stress Conditions

In this study, 20,133,859 SNPs were used to perform the GWAS on the GP of the 180 okra accessions under CK and the two salt treatments (T1 and T2). Using the MLM model from the EMMAX and GEMMA software, 511 SNP loci were significantly associated with six traits (Figure S1). Further statistical analysis of these SNPs revealed that 167 were detected by both programs simultaneously, and they are relatively stable SNP loci (Table S5). These 167 SNPs were located on 21 of the 65 okra chromosomes, namely, chromosomes 4, 5, 15, 17, 19, 22, 24, 25, 31, 37, 40, 41, 43, 44, 45, 46, 54, 56, 59, 61, and 64. Among them, chromosome 31 had the most marker–trait associations (66) detected. Four SNPs were detected for GP-CK-3 d, and three SNPs were detected for GP-CK-7 d under the CK condition. Under the salt treatment conditions, ten SNPs were detected for GP-T1-3 d, 90 SNPs were detected for GP-T1-7 d, 36 SNPs were detected for GP-T2-3 d, and 24 SNPs were detected for GP-T2-7 d. More SNPs for the four traits were detected after the salt treatment than for the CK, which may be related to the wider range of phenotypic variations in the population (Table 1).

Among the 167 SNPs, the majority were only detected in one trait. SNP10374331 was located on chromosome 41 and was detected simultaneously in GP-T1-7 d and GP-T2-7 d, and was a stable locus associated with GP on day 7 after the salt treatment. Additionally, two SNPs caught our attention, namely, SNP2619493 on chromosome 59 and SNP2692266 on chromosome 44. SNP2619493 was detected simultaneously in GP-CK-3 d, GP-T1-3 d, and GP-T2-3 d, while SNP2692266 was detected simultaneously in GP-CK-7 d, GP-T1-7 d, and GP-T2-7 d (Table 3), indicating that these two SNPs were stable under CK and the two salt treatments that were used in this study, and that they were important SNP loci for regulating the GP of okra seeds.

### 2.4. Candidate Gene Predictions

**Figure 4:**
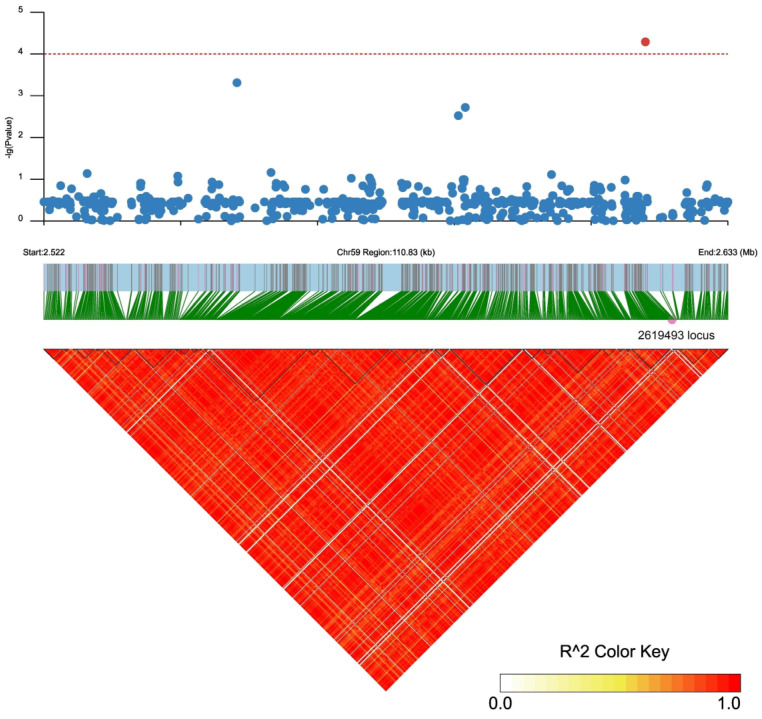
LD block analysis of SNP2619493 on chromosome 59. Regional Manhattan plot (**top**) and LD heatmap (**bottom**) surrounding the SNP2619493. Red dotted line represents the significance threshold of −log10 (*p*) = 4. Red circle indicates SNP2619493, and blue circles indicate the SNP loci that are not significantly associated. Triangles indicate individual haplotype blocks.

**Figure 5:**
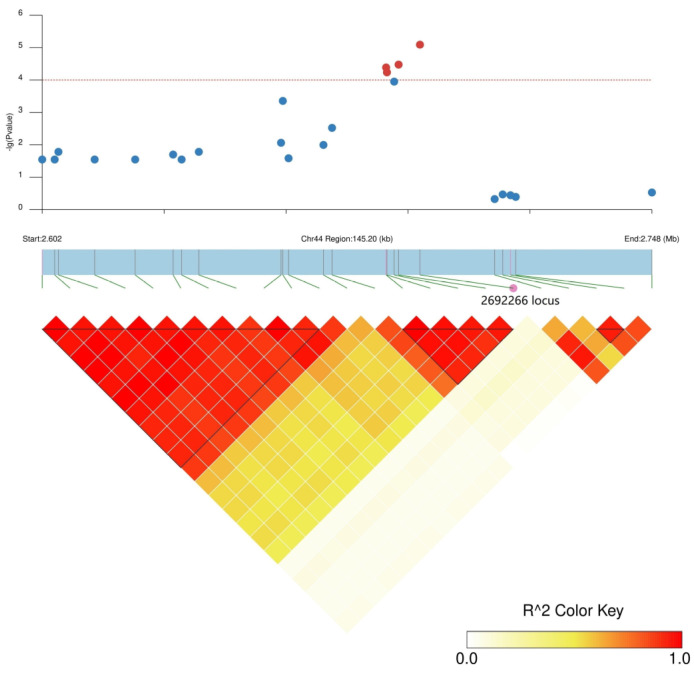
LD block analysis of SNP2692266 on chromosome 44. Regional Manhattan plot (**top**) and LD heatmap (**bottom**) surrounding the SNP2692266. Red dotted line represents the significance threshold of −log10 (*p*) = 4. Red circles indicate SNP2692266, and blue circles indicate the SNP loci that are not significantly associated. Triangles indicate individual haplotype blocks.

There were 31 and 36 annotated genes in the upstream and downstream 100 kb range of SNP2619493 on chromosome 59 and SNP2692266 on chromosome 44, respectively (Table S6). We conducted LD block analysis on the two SNPs to further screen for candidate genes near SNP2619493 and SNP2692266 (Figure 4 and Figure 5). The left side of SNP2619493 was a larger LD block (LD block 29: SNP2616096–SNP2618895), the right side was a very small LD block (LD block 30: SNP26197510-SNP2619815), and SNP2619493 was not within any LD block (Figure 4; Table S7). SNP2692266 was within a larger LD block (LD block 2: SNP2686119–SNP2692266) (Figure 5; Table S7).

**Figure 6:**
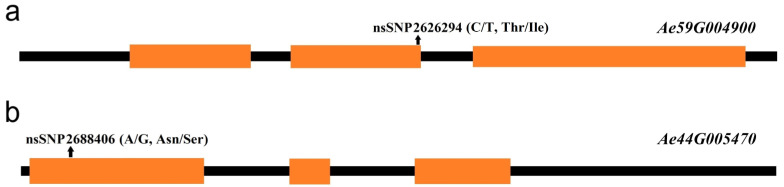
The position of the nsSNPs in the candidate genes. (**a**) The position of nsSNP2626294 in the *Ae59G004900* gene; (**b**) the position of nsSNP2688406 in the *Ae44G005470* gene. Orange squares represent the exon regions, and black lines represent the noncoding regions.

**Figure 7:**
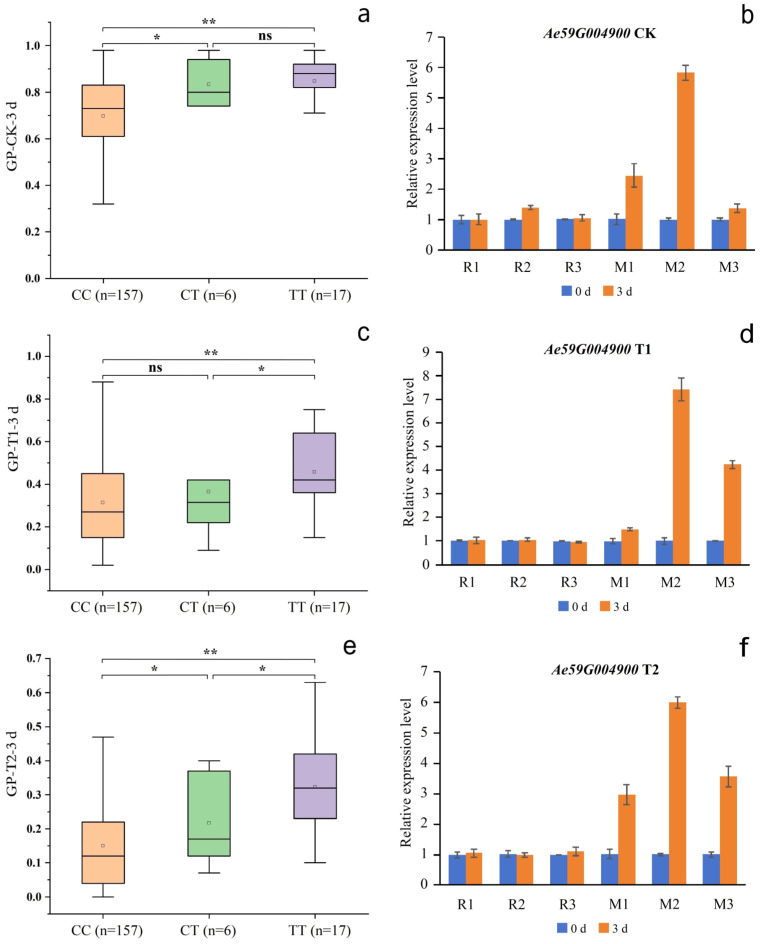
Phenotypes of the accessions with different nsSNP2626294 in *Ae59G004900* alleles. (**a**,**c**,**e**) represent the GP-CK-3 d, GP-T1-3 d, and GP-T2-3 d of the 180 accessions, respectively; CC represents the homozygous reference allele, CT represents the heterozygous allele, TT represents the homozygous mutant allele; n represents the number of accessions; * and ** represent significant differences at *p* < 0.05 and *p* < 0.01 by the *t*-test, respectively. (**b**,**d**,**f**) represent the gene expression levels of *Ae59G004900* under the CK, T1, and T2 conditions, respectively. W1, W2, and W3 represent the CC-type accessions 18, 34, and 70, respectively; M1, M2, and M3 represent the TT-type accessions 32, 99, and 157, respectively.

Although SNP2619493 on chromosome 59 was not included in any of the LD blocks, the *Ae59G004900* gene 4.39 kb downstream caught our attention. This gene was located within LD blocks 32 and 33, while SNP2619493 was adjacent to LD block 30, indicating that SNP2619493 was very close to the *Ae59G004900* gene. *Ae59G004900* was located between 2,623,883 and 2,628,492 bp on the chromosome. A nsSNP2626294 (C/T) was detected at 1536 bp of the CDS sequence of this gene, which caused the encoded amino acid to change from threonine (Thr, ACC) to isoleucine (Ile, ATC) (Figure 6a). Among the 180 okra accessions, 157 (CC) did not undergo mutations in nsSNP2626294, 17 accessions (TT) underwent homozygous mutations, and 6 accessions (CT) underwent heterozygous mutations (Figure 7a). The statistical analysis of the phenotypic data of these three types of accessions revealed that the GP-CK-3 d of the 17 TT-type accessions and 6 CT-type accessions were significantly higher than that of the 157 CC-type accessions. The GP-T1-3 d of the TT-type accessions was significantly higher than that of the CT-type accessions and the CC-type accessions, and the GP-T1-3 d of the CT-type accessions was higher than that of the CC-type accessions. The GP-T2-3 d of the TT-type and CT-type accessions were significantly higher than that of the CC-type accessions (Figure 7a,c,e). The amino acid changes caused by nsSNP2626294 (C/T) in *Ae59G004900* led to an increase in phenotypic values, suggesting that *Ae59G004900* may regulate the GP of okra seeds.

**Figure 8:**
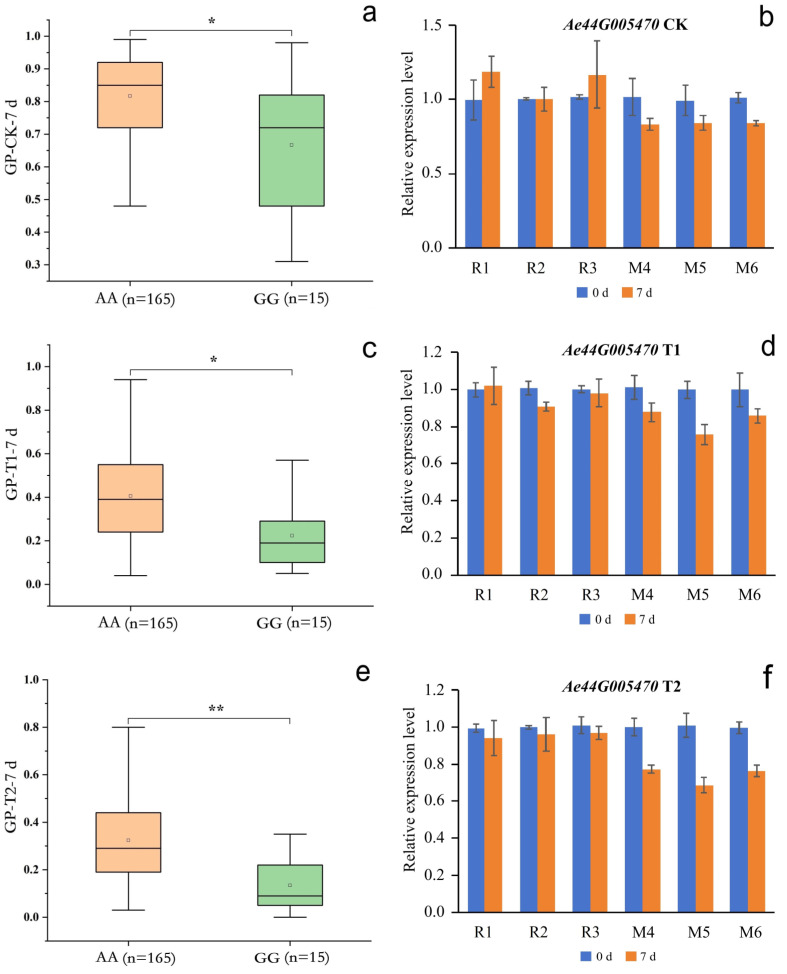
Phenotypes of the accessions with different nsSNP2688406 in *Ae44G005470* alleles. (**a**,**c**,**e**) represent the GP-CK-7 d, GP-T1-7 d, and GP-T2-7 d of the 180 accessions, respectively; AA represents the homozygous reference allele, and GG represents the homozygous mutant allele; n represents the number of accessions; and * and ** represent significant differences at *p* < 0.05 and *p* < 0.01 by the *t*-test, respectively. (**b**,**d**,**f**) represent the gene expression levels of *Ae44G005470* under the CK, T1, and T2 conditions, respectively. W1, W2, and W3 represent the AA-type accessions 18, 34, and 70, respectively; M4, M5, and M6 represent the GG-type accessions 66, 82, and 141, respectively.

SNP2692266 on chromosome 44 was located within an LD block, and this LD block contained five SNPs. SNP2688406 was an nsSNP, adjacent to SNP2692266. nsSNP2688406 was located at 60 bp of the *Ae44G005470* CDS sequence, causing a mutation from A to G, resulting in an amino acid change from asparagine (Asn, AAT) to serine (Ser, AGT) (Figure 6b). Among the 180 okra accessions, 165 (AA) did not undergo mutations in nsSNP2688406, 15 accessions (GG) underwent homozygous mutations, and no accession (CT) underwent a heterozygous mutation (Figure 8a). The GP-CK-7 d of the GG-type accessions was significantly lower than that of the AA-type accessions, the GP-T1-7 d of the GG-type accession was significantly lower than that of the AA-type accessions, and the GP-T2-7 d of the GG-type accession was significantly lower than that of the AA-type accessions (Figure 8a,c,e). The amino acid changes caused by nsSNP2688406 (A/G) in *Ae44G005470* led to a decrease in phenotypic values, suggesting that *Ae44G005470* may regulate the GP of okra seeds.

### 2.5. Candidate Gene Expression Analysis

To further verify the functions of *Ae59G004900* and *Ae44G005470* in regulating the GP, okra accessions 18 (R1), 34 (R2), 70 (R3), 32 (M1), 99 (M2), 157 (M3), 66 (M4), 82 (M5), and 141 (M6) were used as materials to conduct the qRT-PCR analysis. The genotype of nsSNP2626294 of *Ae59G004900* in R1, R2, and R3 was CC, which was a homologous reference allele. The genotype of nsSNP2626294 in M1, M2, and M3 was TT, which was a homozygous mutant allele. The genotype of nsSNP2688406 of *Ae44G005470* in R1, R2, and R3 was AA, which was a homologous reference allele. The genotype of nsSNP2688406 in M4, M5, and M6 was GG, which was a homozygous mutant allele (Figure 7 and Figure 8).

Under the CK, T1, and T2 conditions, *Ae59G004900* in R1, R2, and R3 was not significantly differentially expressed on days 0 or 3 (Figure 7b,d,f), indicating that the reference genotype of this gene did not respond during seed germination. The expression level of *Ae59G004900* in M1, M2, and M3 was significantly higher on day 0 than on day 3, indicating that the nsSNP2626294 mutation in this gene is involved in the positive regulation of seed germination. No significant differential expression of *Ae44G005470* in R1, R2, and R3 on days 0 or 7 was detected under the CK, T1, or T2 conditions (Figure 8b,d,f), indicating that the reference genotype of this gene did not respond during seed germination. The expression level of *Ae44G005470* in M4, M5, and M6 on day 7 was significantly lower than that on day 0, indicating that the nsSNP2688406 mutant of this gene is involved in the negative regulation of seed germination. Therefore, we speculate that *Ae59G004900* and *Ae44G005470* play a crucial role in regulating the germination process in okra seeds. *Ae59G004900* is annotated as “cellulose synthase” in the GO, KEGG, Pfam, Swissprot, TrEMBL, and nr databases. *Ae44G005470* is annotated as “cellular components: integral component of the membrane” in the GO analysis and as “phytosulfokine protein (PSK)” in the Pfam, Swissprot, and TrEMBL databases.

## 3. Discussion

### 3.1. GP of Okra Seeds under Salt Stress

Okra has low seed vitality, which is mainly manifested by a low germination rate, long germination time, uneven emergence, and poor stress resistance, which are the main obstacles in cultivating and producing okra [36,37]. Under suitable germination conditions, the GP of Marsaouia okra is 88.0%, but the emergence percentage is only 64% [38]. The GP of Hisar Unnat okra is 66.3% [39]. Under seed priming conditions, the GPs of six okra accessions were 25.33–88.00% [40]. In this study, the 180 okra accessions had a GP of 80.41% on day 7 under the CK condition, while the GP on day 3 was only 70.81%. The overall GP of the 180 okra accessions was low, which may be related to the thicker seed coat, the presence of hard seeds, or the strong dormancy of okra [41,42]. Seed germination is a critical stage during plant growth and development, as well as one of the most salt-sensitive stages. Salt stress has a significant inhibitory effect on seed germination [43]. The GP of okra decreases further when seeds encounter adverse conditions, such as salt and a low temperature [38].

The GPs of the 180 okra accessions in this study decreased significantly under the two salt concentrations compared to the CK. GP-T1-3 d was 32.94%, with a decrease of 114.97%, and GP-T1-7 d was 39.07%, with a decrease of 105.81%. GP-T2-3 d was 16.64%, with a decrease of 326.12%, and GP-T2-7 d was 30.77%, with a decrease of 161.33%. The average GP of 295 rice accessions [44], 191 soybean accessions [24], 96 cotton accessions [14], 211 camelina accessions [45], and 520 rape accessions [46] under 200 mM, 150 mM, 200 mM, 100 mM, and 230 mM NaCl treatments were 87.00%, 52.59%, 45.3%, 78.31%, and 36.92%, respectively. These results show that the salt tolerance of okra during seed germination is low compared with food and cash crops. Therefore, improving the GP of okra seeds under salt stress is particularly important for enhancing the salt tolerance of okra.

### 3.2. Mining the Candidate Genes Regulating the GP of Okra Seeds under Salt and No-Salt Stresses

MLM is widely used in GWASs due to its relatively high accuracy and rigor [47,48]. Many millions of SNPs are being used for GWASs, which will increase the time and difficulty of MLM operations. Thus, the faster and more accurate EMMAX [49] and GEMMA [50] algorithms have been developed. GWASs using EMMAX and GEMMA have been adopted simultaneously in many studies to compensate for their respective shortcomings [51,52,53]. This study used 20,133,859 SNPs to perform a GWAS on 180 okra accessions under the CK and two salt treatments. Using the MLM model from EMMAX and GEMMA software, 511 significantly correlated SNPs were detected. Among them, EMMAX detected 201 and GEMMA detected 310. Therefore, GEMMA is superior to EMMAX in terms of SNP detection efficiency, which is consistent with the results of Liu et al. [53]. The simultaneous use of the two programs will help improve the accuracy of GWASs and obtain relatively reliable SNP loci. Thus, we used the 167 SNPs that were detected by both programs as reliable SNPs.

Among the 167 SNPs that were detected simultaneously using EMMAX and GEMMA, SNP2619493 on chromosome 59 was detected simultaneously in GP-CK-3 d, GP-T1-3 d, and GP-T2-3 d, while SNP2692266 was detected simultaneously on chromosome 44 in GP-CK-7 d, GP-T1-7 d, and GP-T2-7 d, indicating that these two SNPs are associated with low-salt-stress, high-salt-stress, and no-salt-stress conditions and are stable. Finding stable SNP loci is an important step in a GWAS; however, there were 31 and 36 annotated genes in the upstream and downstream 100 kb range of SNP2619493 and SNP2692266, respectively. Therefore, identifying candidate genes from these annotated genes is another important task. Methods based on LD block analysis are increasingly being applied to identify candidate genes [13,15,16]. The LD block region is usually considered a confidence interval for a candidate gene [54]. Our LD block analysis revealed that SNP2619493 was not within any LD block but was sandwiched between LD block 29 and LD block 30 (Figure 4; Table S7). Only two SNPs were detected between these two LD blocks, SNP2619136 and SNP2619493. The key SNP locus that was detected in the three traits was not within an LD block, which may have been caused by missing data points or incorrect sequence assembly in a particular region during sequencing, which interrupted the haplotype block [17]. We inferred that there must be a key candidate gene near SNP2619493, so we screened for genes near this SNP. We identified the *Ae59G004900* gene located 4.39 kb downstream of SNP2619493, which contained an nsSNP2626294 (C/T) within its CDS. nsSNP2626294 caused the amino acid that is encoded by *Ae59G004900* to change from Thr to Ile, leading to an increase in phenotypic values in 17 homozygous mutant allele accessions. This result further confirms that *Ae59G004900* is the candidate gene, and nsSNP2626294 is a key molecular marker. In some cases, important SNPs are not within specific haplotype blocks, but further analysis is needed to confirm their effectiveness. Our GWAS provided a region related to the traits, and we needed to identify the candidate genes within this region.

### 3.3. Functions of PSK and ACF Genes under Salt Stress

We determined that *Ae59G004900* and *Ae44G005470* play important regulatory roles in okra seed germination. *Ae59G004900* was annotated as “cellulose synthase”, and *Ae44G005470* was annotated as the “phytosulfokine (PSK) protein”. Cellulose is the main component of the primary and secondary cell walls, and its synthesis is mainly catalyzed and regulated by cellulose synthase (CESA). CESA genes usually exist in the form of gene families, and their main function is to regulate the synthesis of primary and secondary cell walls [55]. Studies have shown that some CESA genes are also involved in the regulation of salt stress in plants. Under salt stress conditions, *CESA6* plays a major role in cellulose deposition in Arabidopsis roots, and sustained cellulose synthesis is crucial for salt tolerance. When the *CESA6* mutation leads to increased sensitivity to salt stress, it affects the response of downstream stress genes [56]. The *CesA1* RNAi-silenced broccoli plants have enhanced salt tolerance. Although these silenced plants exhibit lower cellulose and pectin contents, they have higher soluble sugar and proline contents [57]. Applying NaCl stress may stimulate the expression of some CESA genes in cucumbers [58]. In this study, the expression level of the *Ae59G004900* gene containing the nsSNP2626294 mutant increased under salt stress during seed germination, demonstrating the function of CESA genes in regulating salt tolerance in okra. PSK represents a group of plant peptide growth factors that are ubiquitously present in higher plants and have universal functions [59]. The PSK genes participate in regulating disease resistance under stress [60], drought [61], and osmotic stress [62]. In this study, the *Ae44G005470* gene negatively regulated the salt tolerance of okra seeds during germination, indicating that PSK genes also function in regulating salt tolerance in plants.

In this study, we identified *Ae59G004900* and *Ae44G005470* related to salt tolerance during okra germination. As the tolerance of crops to salt stress varies at different developmental stages, it is important to study the salt-tolerant functions of these two genes at other developmental stages. However, the SNP loci nsSNP2626294 and nsSNP2688406 that were identified in this study may have potential application value in salt-tolerant okra breeding, as we discovered the salt-tolerant phenotypic variations in some okra accessions and the gene expression changes that are caused by them. Breeders can use these two SNP markers for molecular-marker-assisted selection of salt tolerance in okra, particularly for salt tolerance during germination. Research on salt tolerance in okra at the genetic level is very limited, and this study lays the foundation for the genetic improvement of salt tolerance in okra. In addition, our results enhance our understanding of the inheritance of salt tolerance in okra. However, more research is needed to reveal the genetic mechanism of salt tolerance in okra.

## 4. Materials and Methods

### 4.1. Plant Materials

A total of 180 okra accessions were collected worldwide for the current study. The majority (115) was from China, while the remaining accessions were from Japan (25), USA (9), India (6), Brazil (4), Thailand (4), France (3), Bangladesh (2), Malaysia (2), Nigeria (2), the Philippines (2), Canada (1), Vietnam (1), Egypt (1), Mali (1), Kenya (1), and Ethiopia (1) (Table S1). All accessions were provided by National Crop Germplasm Resource Bank of China National Science and Technology Resource Sharing Service Platform (Vegetable and flower branch bank).

### 4.2. Seed Germination Experiment

All accessions were planted in 2022 at the Jiangsu Yanjiang Institute of Agricultural Sciences (Nantong city, 120°57′ E, 32°37′ N). The seeds were harvested at full maturity, and the seeds from all accessions were placed in a 45 °C oven for 3 days to break dormancy. A germination experiment was conducted under salt stress using the seeds of the 180 okra accessions. Mature, full, and relatively consistently sized seeds were selected, and placed in a 9 cm Petri dish containing 0 mmol/L NaCl (CK, diluted water), 70 mmol/L NaCl (treatment 1, T1), or 140 mmol/L NaCl (treatment 2, T2) solutions for germination. Each Petri dish contained 50 seeds, and 2.5 mL of solution was added to each dish. The experiment was repeated three times. The Petri dishes were padded with filter paper to maintain a damp environment. The dishes containing seeds were placed in an incubator at a constant temperature of 25 °C. The solution in the dishes was replaced every day. On days 3 and 7 of incubation, germination was investigated [14], and the germination standard was based on the germ exceeding 2 mm in length. The germination status of 180 okra seeds was evaluated using the germination percentage (GP) as an indicator.

GP (%) = number of germinated seeds/number of total seeds × 100%

The GP of the CK condition on days 3 and 7 (GP-CK-3 d and GP-CK-7 d), the T1 condition on days 3 and 7 (GP-T1-3 d and GP-T1-7 d), and the T2 condition on days 3 and 7 (GP-T2-3 d and GP-T2-7 d) were used as the phenotypic data to perform the GWAS with the SNP data of the 180 okra accessions.

### 4.3. DNA Extraction and Whole-Genome Resequencing

Young leaves of each accession were cut and ground into a powder in liquid nitrogen. Genomic DNA was extracted using the Plant Genomic DNA Kit (TIANGEN Biotech, Beijing, China). The DNA solution was treated with RNase and stored at −80 °C. Agarose gel electrophoresis (0.8%) was used to detect the quality of the DNA, and the NanoDrop 2000 spectrophotometer (Thermo Fisher Scientific, Wilmington, DE, USA) was used to detect the DNA concentration.

Genomic DNA was used to construct the library following the manufacturer’s instructions (Illumina, San Diego, CA, USA). After constructing the DNA library, sequencing was performed on the Illumina HiSeq X Ten platform with 150 bp read lengths by Biomarker Technologies Corp. (Beijing, China). Raw reads were filtered based on the following criteria: pair-end reads with >10%, “N” bases, and reads in which >50% of the bases had a quality score <20 (Phred-like score). The high-quality sequences were obtained for subsequent analyses.

All clean reads for each accession were mapped to the Okra Wufu reference genome (NCBI BioProject: PRJNA971663) using Baw-mem v2.2.1 (https://github.com/bwa-mem2/bwa-mem2, accessed on 10 October 2022) with default parameters. SNPs were called using the HaplotypeCaller module in GATK (v3.8) [63] and were filtered using the following parameters: QD < 2.0 || MQ < 40.0 || FS > 60.0 || QUAL < 30.0 || MQrankSum < −12.5 || ReadPosRankSum < −8.0 -clusterSize 2 clusterWindowSize 5. The SNPs were annotated based on the Wufu reference genome using snpEff software (3.6 c) [64].

### 4.4. GWAS Analysis

Principal component analysis (PCA) was performed using the smartPCA program in EIGENSOFT software [65]. The maximum likelihood method in the ADMIXTURE program was used to evaluate the population structure through 100,000 Markov Chain Monte Carlo iterations [66]. The number of subgroups (K) was set from 1 to 10, and the K value was run five times.

Only SNPs with MAF ≥ 0.05 and missing rate ≤ 0.8 in the population were used to carry out GWAS. The GWAS of the six traits (GP-CK-3 d, GP-CK-7 d, GP-T1-3 d, GP-T1-7 d, GP-T2-3 d, and GP-T2-7 d) was performed using EMMAX (Efficient Mixed-model Association eXpedated) [49] and GEMMA (Genome-wide Efficient Mixed Model Association) [50] software based on the mixed linear model (MLM). The kinship matrix generated with the emmax-kin-intel package of EMMAX was used to correct the population structure. For the two software packages, the first three principal components (PCs) derived from the whole-genome SNPs were fitted as fixed effects to correct the population structure. The threshold for significant association between the SNPs and target traits was −log10 (P) > 4.

GGplot2 software [67] was used to draw the Quantile scatterplot (Q-Q plot), and QQ man software [68] was used to draw the Manhattan plot to display the significant SNP loci in the GWAS. Significantly associated SNPs of the six traits were annotated. The genes within 100 kb upstream and downstream of significantly associated SNP loci were selected for functional annotation in the COG, GO, KEGG, KOG, Pfam, SwissProt, and NR databases.

### 4.5. Identification of Haplotype Blocks and Characterization of Candidate Genes

Linkage disequilibrium (LD) analysis was performed on significantly associated SNP loci using Haploview v4.2 software [69] to obtain the LD blocks. The correlation coefficient (R^2^) was calculated to determine the pairwise LD decay. SNPs within an LD block were usually considered key SNPs that were significantly associated with traits. If SNPs associated with traits were not within a particular LD block, the SNPs or genes within its closest up- and downstream LD blocks should also be considered [17]. Subsequently, we performed comparative analyses of GP in the accessions with different SNPs of the candidate gene associations and further excluded the candidate genes that were obtained from the inaccurate lead SNPs.

We identified the nonsynonymous SNPs (nsSNPs) around the significantly associated SNPs and analyzed the distribution of homozygous reference alleles, heterozygous alleles, and homozygous mutant alleles among the 180 accessions. Then, we compared and analyzed the GPs of the different allele accessions to verify the effect of a change in amino acid on the GPs of different allele accessions caused by the nsSNP.

### 4.6. Quantitative Real-Time Polymerase Chain Reaction (qRT-PCR) Analysis of Candidate Genes

The nsSNP2626294 (within the *Ae59G004900* gene) and nsSNP2688406 (within the *Ae44G005470* gene) of okra accessions 18, 34, and 70 were genotypes in the reference genome, and the GP of 18, 34, and 70 were at a moderate level. Accessions 32, 99, and 157, with higher GPs, were homozygous mutant materials of nsSNP2626294. Accessions 66, 82, and 141, with lower GPs, were the homozygous mutant materials of nsSNP2688406. These nine accessions were used for qRT-PCR analysis to verify the functions of the candidate genes.

Total RNA was extracted from the above nine accessions using TRIzol (Invitrogen, Carlsbad, CA, USA), and RNA quality was detected using the NanoDrop 2000 spectrophotometer (Thermo Scientific, Waltham, MA, USA). RNA was reverse-transcribed into cDNA using the Prime HiFi-MMLV cDNA kit (CWBIO, Beijing, China). The qRT-PCR primers for the candidate genes were designed using Primer Premier 5.0 software. Actin was used as the internal reference gene [70]. The specific primer sequences are listed in Table S2. The 20 µL qRT-PCR system consisted of 0.5 µL UltraSYBR EnzymeMix (CWBIO), 0.5 µL each of the forward and reverse primers, 1 µL of cDNA template, 10 µL of buffer, and 7.5 µL of ddH_2_O. To obtain the relative expression levels of the candidate genes, the original qRT-PCR data were analyzed by the 2^−ΔΔCt^ method [71].

## 5. Conclusions

In summary, we identified the seed germination-related genes *Ae59G004900* and *Ae44G005470* in okra through a GWAS under low-salt-stress (T1), high-salt-stress (T2), and no-salt-stress (CK) conditions. nsSNP2626294 and nsSNP2688406 were in the coding regions of the two genes and led to an increase and a decrease in the GP of okra, respectively. We infer that *Ae59G004900* and *Ae44G005470* are key genes regulating the GP of okra, and both genes play a regulatory role under no-salt and salt stress conditions. After further screening the okra materials by using the specific molecular markers and functional verification by transgene, these two SNP markers and two genes can be applied in molecular breeding of okra seed germination under salt and no-salt stresses, whether in the laboratory or in the field.

## Figures and Tables

**Table 1:** Phenotypic variation in GPs of the 180 okra accessions.

Trait	Mean	Max	Min	SD	CV (%)	Skew.	Kurt.
GP-CK-3 d	0.7081	0.9800	0.1000	0.1836	25.92	−0.9932	0.9420
GP-T1-3 d	0.3294	0.9333	0.0200	0.2069	62.82	0.6535	−0.1401
GP-T2-3 d	0.1664	0.6333	0.0000	0.1366	82.07	0.9112	0.2628
GP-CK-7 d	0.8041	0.9933	0.3067	0.1445	17.96	−1.0539	0.8917
GP-T1-7 d	0.3907	0.9400	0.0467	0.2022	51.76	0.3450	−0.6176
GP-T2-7 d	0.3077	0.9200	0.0000	0.1841	59.82	0.5847	−0.0123

GP-CK-3 d: GP of CK condition on day 3. GP-CK-7 d: GP of CK condition on day 7. GP-T1-3 d: GP of T1 condition on day 3. GP-T1-7 d: GP of T1 condition on day 7. GP-T2-3 d: GP of T2 condition on day 3. GP-T2-7 d: GP of T2 condition on day 7. SD: standard deviation. CV: coefficient of variation. Skew.: skewness. Kurt.: kurtosis.

**Table 2:** Correlations between GPs under CK, T1, and T2 conditions in 180 okra accessions.

	GP-CK-3 d	GP-CK-7 d	GP-T1-3 d	GP-T1-7 d	GP-T2-3 d	GP-T2-7 d
**GP-CK-3 d**						
**GP-CK-7 d**	0.6287 **					
**GP-T1-3 d**	0.3863 **	0.2635 **				
**GP-T1-7 d**	0.3637 **	0.2879 **	0.6651 **			
**GP-T2-3 d**	0.3082 **	0.1825 *	0.6719 **	0.5297 **		
**GP-T2-7 d**	0.3800 **	0.3164 **	0.5741 **	0.7069 **	0.6195 **	

GP-CK-3 d: GP of CK condition on day 3. GP-CK-7 d: GP of CK condition on day 7. GP-T1-3 d: GP of T1 condition on day 3. GP-T1-7 d: GP of T1 condition on day 7. GP-T2-3 d: GP of T2 condition on day 3. GP-T2-7 d: GP of T2 condition on day 7. *, **: significant correlation at the 0.05 and 0.01 levels, respectively.

**Table 3:** Summary of the two key SNP loci significantly associated with GP based on the MLM using EMMAX and GEMMA software.

SNP Name	Chromosome	Trait	Software	*p* Value	−log10 (*p*)	Allele	Count	MAF
SNP2619493	Chr. 59	CK-3 d	EMMAX	1.71 × 10^−5^	4.77	A/T	A: 178, T: 160	0.47
			GEMMA	4.67 × 10^−6^	5.33	A/T	A: 178, T: 160	0.47
		T1-3 d	EMMAX	5.12 × 10^−5^	4.29	A/T	A: 178, T: 160	0.47
			GEMMA	1.69 × 10^−5^	4.77	A/T	A: 178, T: 160	0.47
		T2-3 d	EMMAX	7.51 × 10^−5^	4.12	A/T	A: 178, T: 160	0.47
			GEMMA	7.51 × 10^−5^	4.12	A/T	A: 178, T: 160	0.47
SNP2692266	Chr. 44	CK-7 d	EMMAX	2.45 × 10^−5^	4.61	A/G	A: 311, G: 47	0.13
			GEMMA	1.08 × 10^−5^	4.97	A/G	A: 311, G: 47	0.13
		T1-7 d	EMMAX	4.81 × 10^−5^	4.32	A/G	A: 311, G: 47	0.13
			GEMMA	1.27 × 10^−5^	4.90	A/G	A: 311, G: 47	0.13
		T2-7 d	EMMAX	8.11 × 10^−6^	5.09	A/G	A: 311, G: 47	0.13
			GEMMA	4.36 × 10^−6^	5.36	A/G	A: 311, G: 47	0.13

Allele: the left side of the “/” represents the main allele, and the right side represents the secondary allele. Count: allele count. MAF: minor allele frequency.

## Data Availability

The data will be made available upon specific request to the authors. The data are not publicly available due to privacy.

## Supplementary Materials

The following supporting information can be downloaded at https://www.mdpi.com/article/10.3390/plants13050588/s1: Figure S1: Manhattan plots and quantile–quantile (Q-Q) plots of GWAS for GP in okra; Table S1: Origin and classification based on resequencing of 180 okra accessions; Table S2: Sequences of specific primers for two candidate genes; Table S3: The GP of the top ten and bottom ten accessions in each treatment; Table S4: Information on genome resequencing data of 180 okra accessions; Table S5: SNP loci detected by both EMMAX and GEMMA software simultaneously; Table S6: Genes and annotation information on SNP2619493 on chromosome 59 and SNP269226 on chromosome 44; Table S7: LD Information on LD blocks within a 100 kb range upstream and downstream of SNP2619493 and SNP269226.
