# Supplementary material for: Identification of Single Nucleotide Polymorphic Loci and Candidate Genes for Seed Germination Percentage in Okra under Salt and No-Salt Stresses by Genome-Wide Association Study
Source: Plants (Basel). 2024 Feb 22;13(5):588. doi: 10.3390/plants13050588 (PMC10934433; doi:10.3390/plants13050588)
Supplement: Supplementary file 1 [file plants-13-00588-s001.zip › Supplementary figures-final version.pdf]

a

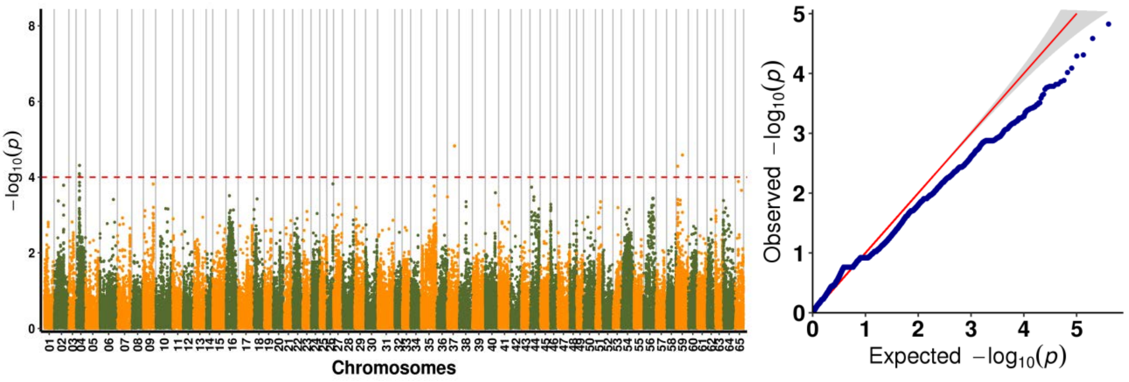

$-\log_{10}(p)$
Chromosomes
Observed $-\log_{10}(p)$
Expected $-\log_{10}(p)$

b

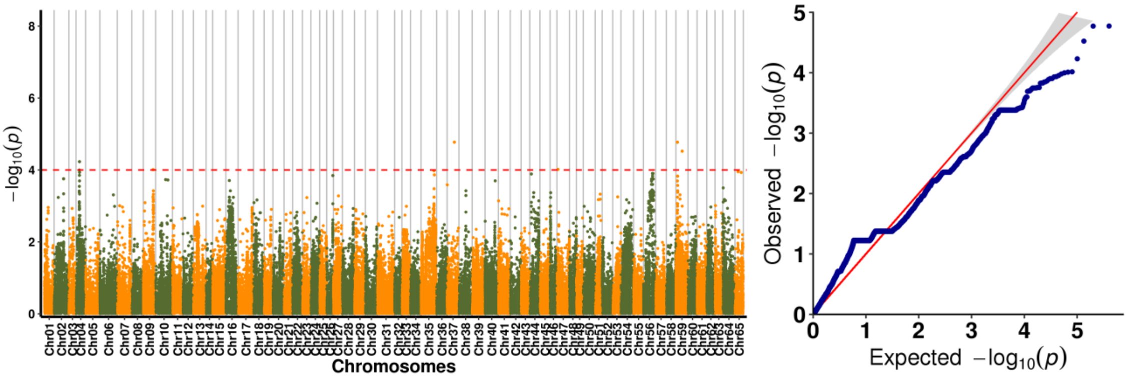

$-\log_{10}(p)$
Chromosomes
Observed $-\log_{10}(p)$
Expected $-\log_{10}(p)$

c

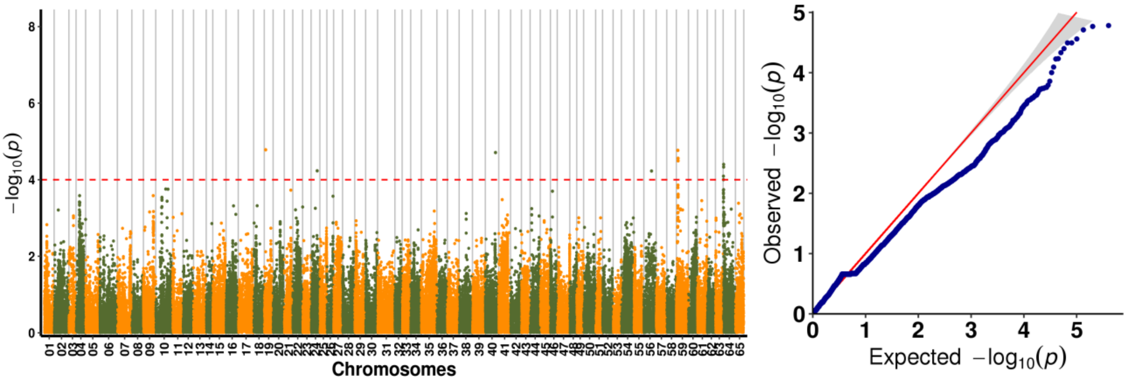

$-\log_{10}(p)$
Chromosomes
Observed $-\log_{10}(p)$
Expected $-\log_{10}(p)$

d

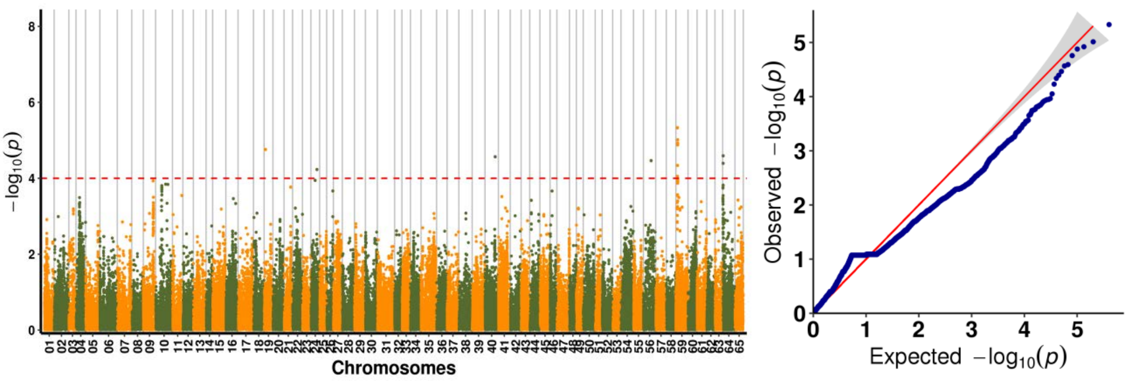

$-\log_{10}(p)$
Chromosomes
Observed $-\log_{10}(p)$
Expected $-\log_{10}(p)$

e

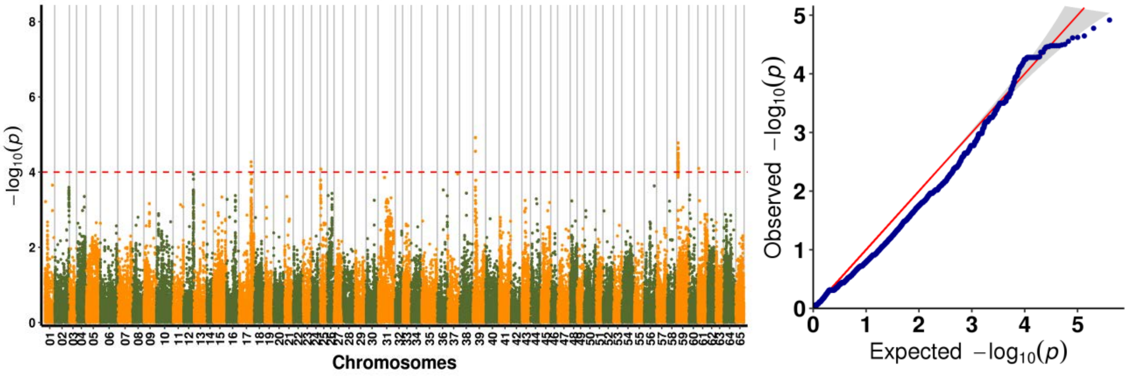

$-\log_{10}(p)$
Chromosomes
Observed $-\log_{10}(p)$
Expected $-\log_{10}(p)$

f

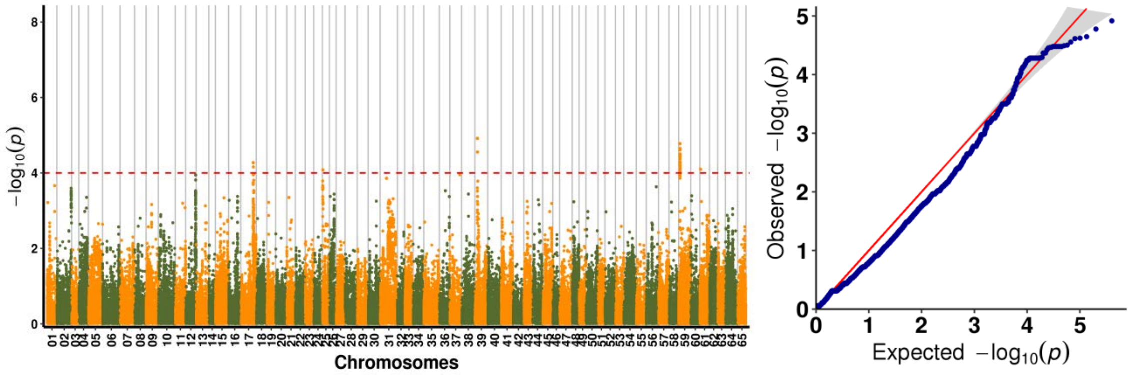

$-\log_{10}(p)$
Chromosomes
Observed $-\log_{10}(p)$
Expected $-\log_{10}(p)$

g

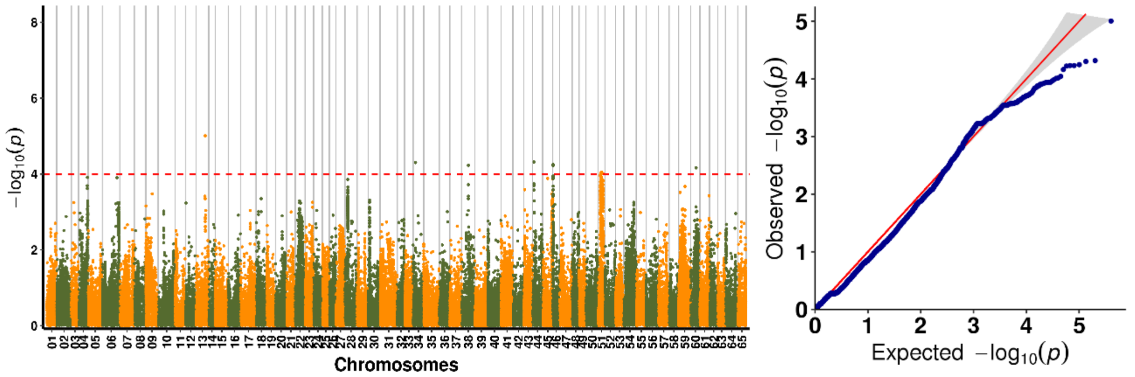

$-\log_{10}(p)$
Chromosomes
Observed $-\log_{10}(p)$
Expected $-\log_{10}(p)$

h

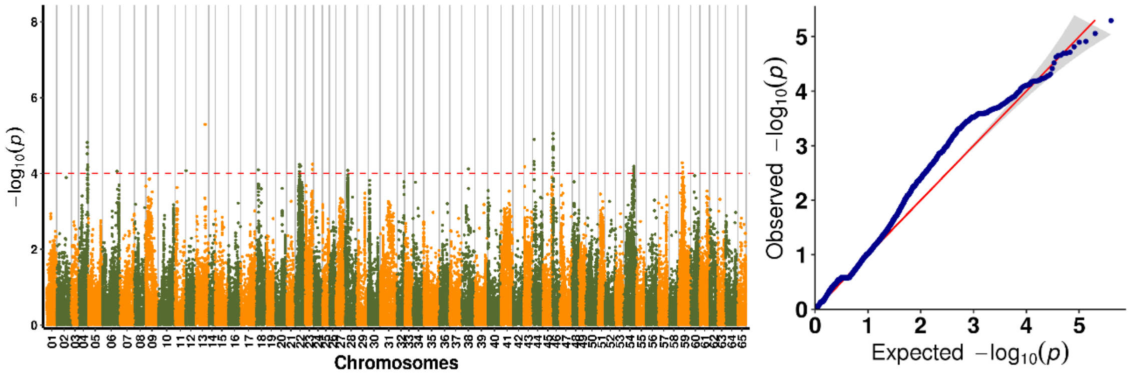

$-\log_{10}(p)$
Chromosomes
Observed $-\log_{10}(p)$
Expected $-\log_{10}(p)$

i

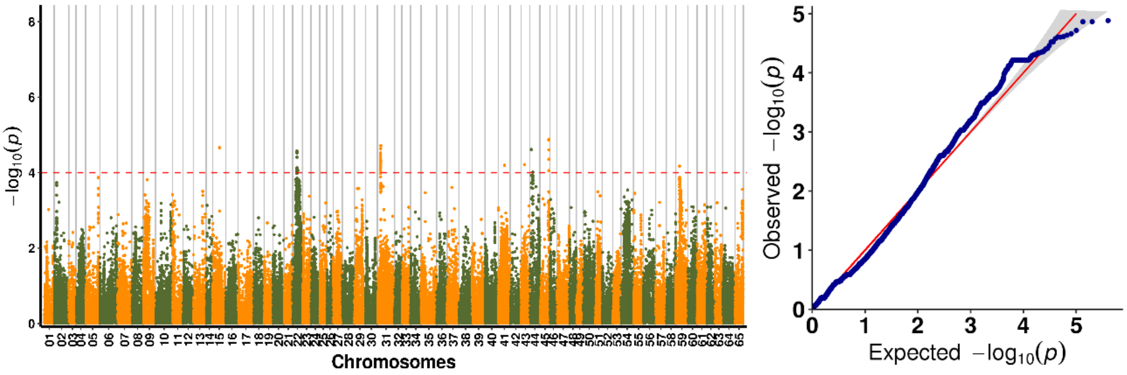
$-\log_{10}(p)$
Chromosomes
Observed $-\log_{10}(p)$
Expected $-\log_{10}(p)$

j

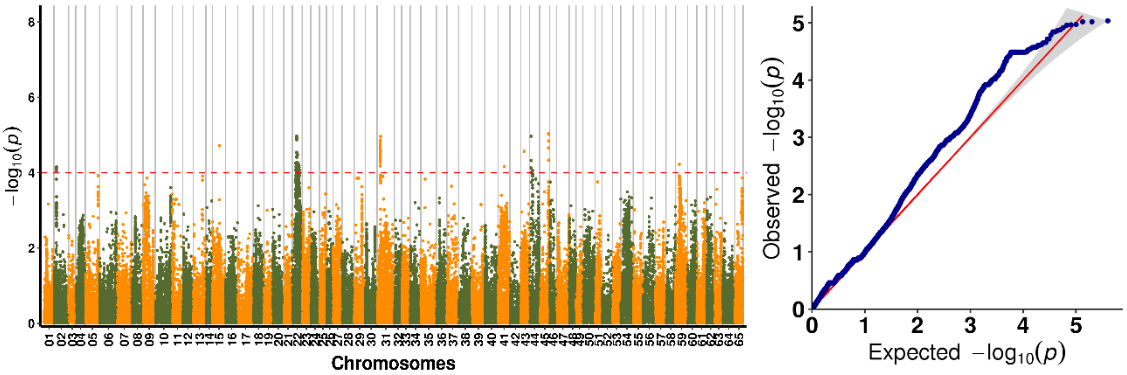
$-\log_{10}(p)$
Chromosomes
Observed $-\log_{10}(p)$
Expected $-\log_{10}(p)$

k

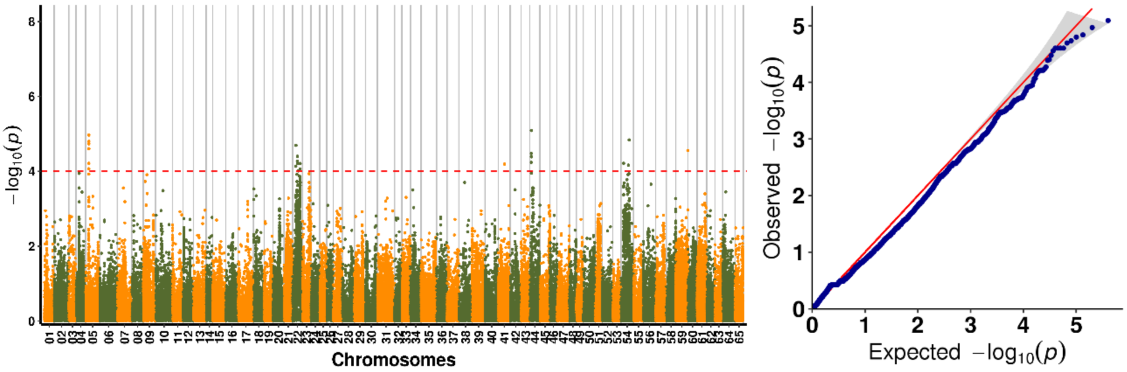
$-\log_{10}(p)$
Chromosomes
Observed $-\log_{10}(p)$
Expected $-\log_{10}(p)$

l

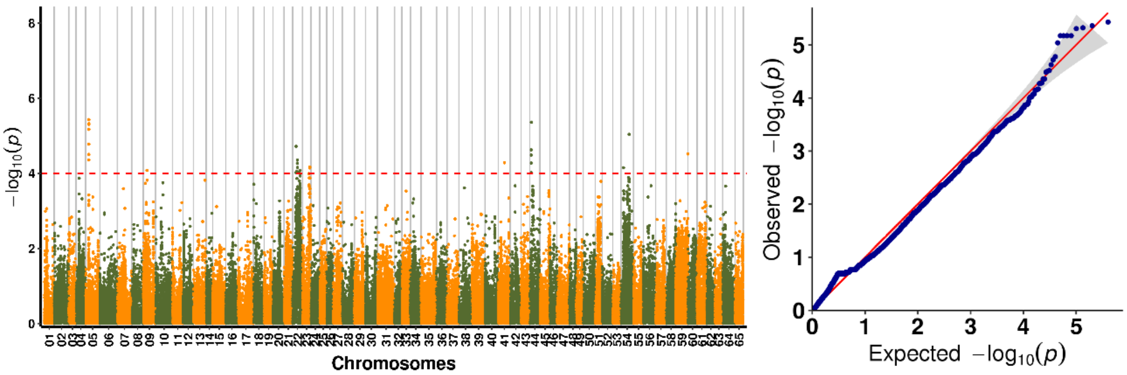
$-\log_{10}(p)$
Chromosomes
Observed $-\log_{10}(p)$
Expected $-\log_{10}(p)$

Figure S1. Manhattan plots and quantile-quantile (Q-Q) plots of GWAS for GP in okra. The threshold line of −log10(P) > 4 (red dashed line) was selected as significant association between SNPs and targeted traits. a: association mapping of GP-CK-3 d based on the EMMAX software; b: association mapping of GP-CK-3 d based on the GEMMA software; c: association mapping of GP-T1-3 d based on the EMMAX software; d: association mapping of GP-T1-3 d based on the GEMMA software; e: association mapping of GP-T2-3 d based on the EMMAX software; f: association mapping of GP-T2-3 d based on the GEMMA software; g: association mapping of GP-CK-7 d based on the EMMAX software; h: association mapping of GP-CK-7 d based on the GEMMA software; i: association mapping of GP-T1-7 d based on the EMMAX software; j: association mapping of GP-T1-7 d based on the GEMMA software; k: association mapping of GP-T2-7 d based on the EMMAX software; l: association mapping of GP-T2-7 d based on the GEMMA software.
